# Potential effects of an invasive seaweed (*Caulerpa cylindracea*, Sonder) on sedimentary organic matter and microbial metabolic activities

**DOI:** 10.1038/s41598-017-12556-4

**Published:** 2017-09-21

**Authors:** Lucia Rizzo, Antonio Pusceddu, Loredana Stabili, Pietro Alifano, Simonetta Fraschetti

**Affiliations:** 1 Department of Biological and Environmental Sciences and Technologies, University of Salento, Via Prov.le Lecce Monteroni, Lecce, Italy; 2 CoNISMa, Piazzale Flaminio, 9, Roma, Italy; 3 Department of Environmental Sciences, Informatics and Statistics, Ca’ Foscari University of Venice, Venice, Italy; 4 Department of Life and Environmental Sciences, University of Cagliari, Via T. Fiorelli 1, Cagliari, Italy; 5 Institute for Coastal Marine Environment of the National Research Council, U.O.S. di Taranto, Via Roma 3, Taranto, Italy

## Abstract

*Caulerpa cylindracea* (Sonder), among the most successful marine bio-invaders on a global scale, poses severe threats to biodiversity. However, the effects of this seaweed on the quantity and the biochemical composition of sedimentary organic matter are still poorly known. Since the whole set of sedimentary features affects the availability of substrates for benthic microbial communities, we: i) investigated the biochemical composition of sediments colonized and not-colonized by *C. cylindracea*, and ii) compared the metabolic patterns of the microbial communities associated with *C. cylindracea* and in the sediments colonized and not-colonized by the seaweed. Our results show that *C. cylindracea* can influence the quantity and biochemical composition of sedimentary organic matter (OM), and that microbial populations associated with colonized sediments do have specific metabolic patterns and degradation capacities. *Caulerpa cylindracea* can also influence the metabolic patterns of the microbial community specifically adapted to degrade compounds released by the seaweed itself, with possible consequences on C cycling.

## Introduction

Biological invasions, a pervasive component of global change^1^, are listed among the most critical threats to biodiversity worldwide^2–4^. The Australian seaweed *Caulerpa cylindracea* (Sonder)^5^ is a non-indigenous species (NIS) now widely distributed in the whole Mediterranean basin^6,7^: its presence alters indigenous shallow benthic assemblages and the metabolism of the fish feeding upon it^8–10^.

High sedimentation rates driven by anthropogenic activities may favour this opportunistic invader^11,12^. In turn, this species, able itself to enhance sediment accumulation, promotes the development of algal turfs^13,14^, compacts layers of sediments up to 15 cm thick, and modifies hydrodynamics near the seabed^15–18^. These features confer *C. cylindracea* to the rank of ecosystem engineer, which, acting with other human-driven threats, is deeply modifying subtidal habitats of the Mediterranean Sea^14^. The ultimate effect of this NIS is a widespread biotic homogenization^12,19^, especially if the invaded habitat is initially less affected by human impacts^20^.

The role of *C. cylindracea* in changing sediment properties is far less known: few studies investigated how *Caulerpa* invasion alters sediment biogeochemical processes and relative ecosystem functions. Sediments invaded by *C. cylindracea* are characterised by higher organic matter (OM) contents and sulphide pools^8,21^, than not invaded areas. In the Adriatic Sea sediments invaded by *C. cylindracea* show organic C, N, P, total protein and carbohydrate contents higher than those in not invaded habitats^22,23^. Sediments that are invaded by *C. cylindracea* can also be characterised by lower C turnover rates than not invaded sediments^23^. Recent efforts, carried out to understand the underlying mechanisms of *C. cylindracea* success and its consequences on the biogeochemistry of the sediments^8,17,18,22,24^, indicate the increase of sedimentary OM quantity as a unique positive effect of *C. cylindracea*.

During the last decades, seaweeds ascribed to the genus *Caulerpa* have invaded different parts of the world, including the Mediterranean, where they are progressively extending their distribution^24^. Some recent studies have pinpointed the potential key role of microbial communities associated with introduced seaweeds in strengthening their capacity to expand and become invasive^25–30^, but mechanisms of this positive interaction are still to be clarified. The ability to transform complex organic macromolecules into low molecular weight compounds through extracellular enzymatic activities makes heterotrophic prokaryotes a key component of marine trophic webs^31^. This also applies to benthic habitats, where the microbial loop^32^ represents a key step in the transfer of detrital OM towards higher trophic levels^33,34^, even acting at very small spatial scales^35^.

Here, we combined the information on the biochemical composition of sedimentary OM (in terms of protein, carbohydrate, lipid and phytopigment contents) with an assessment of the metabolic activities of microbial communities hosted on the seaweed, in colonized and not-colonized sediments. This allowed us an unprecedented view on the effects of *C. cylindracea* on the quantity, composition and potential degradation of sedimentary OM mediated by benthic prokaryotes in coastal marine ecosystems, and their potential consequences on the biogeochemical cycles of sedimentary habitats. More specifically, we hypothesized that the presence of *C. cylindracea* could drive changes in the biochemical composition and the metabolic patterns of microbes. To test this hypothesis, we first assessed differences in the biochemical composition of sedimentary OM among habitats colonized and not-colonized by the seaweed, and then we compared the metabolic patterns of the microbial communities associated with the *C. cylindracea* thalli with those in the sediments colonized and not-colonized by the seaweed. In addition, molecular analyses were performed on algal specimens in order to confirm the identification of the species *C. cylindracea*.

## Results

### DNA extraction, amplification and sequencing of the seaweed

Samples of the seaweed from the different locations were subjected to DNA extraction, PCR amplification and sequencing using *tufA*-specific primer pair. *tufA* amplified sequences were compared to the Genbank database in order to identify the phylogenetic identity of seaweeds from the different collection locations. These analyses confirmed that all of the locations were colonized by the Western Australian species *C. cylindracea* ^5^ (Supplementary Figure S1).

### Biochemical composition of sediments organic matter

**Figure 1:**
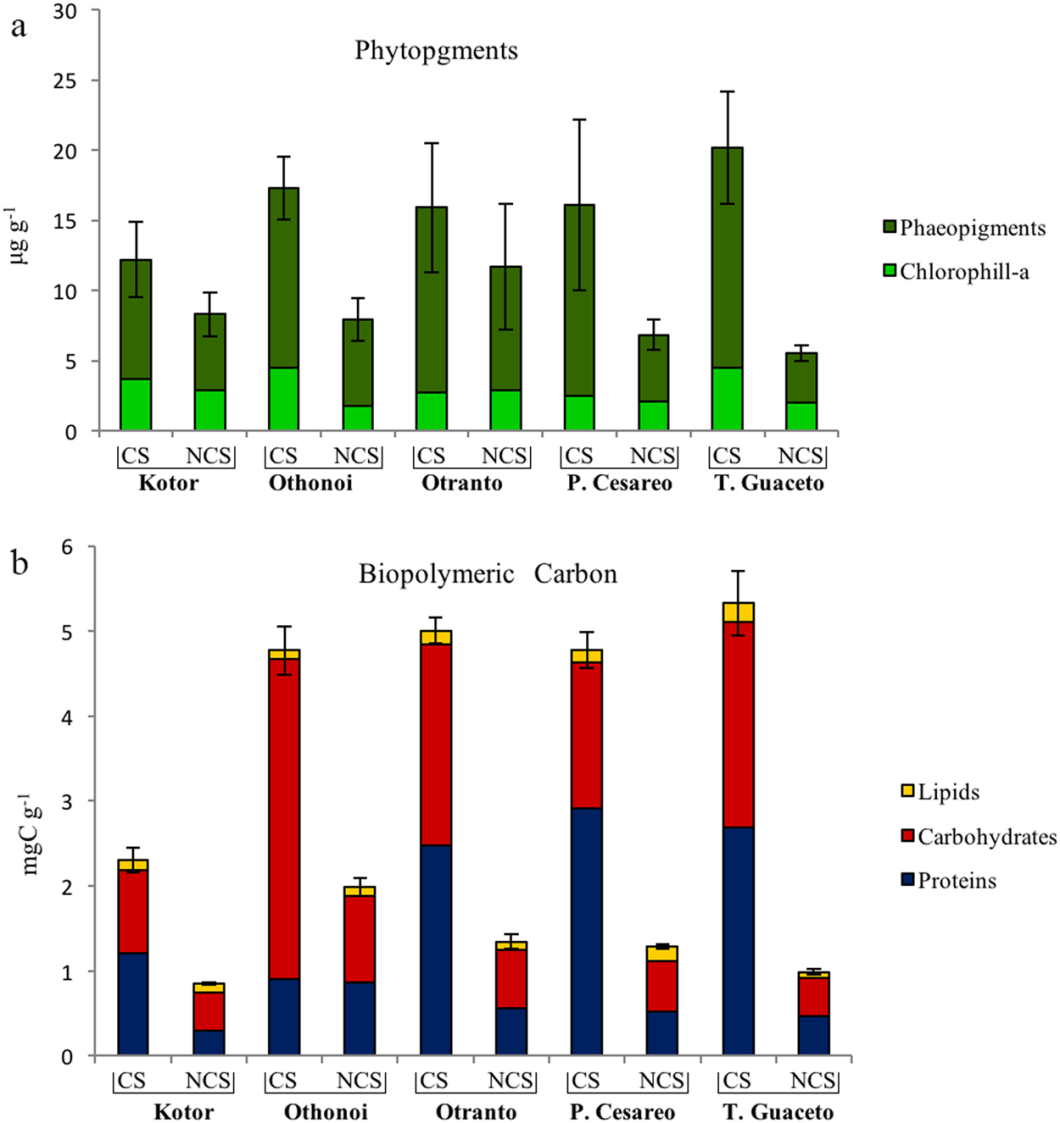
Phytopigment and biopolymeric carbon concentrations in the sediments of the five sampling locations. Reported are concentrations of (**a**) chlorophyll-a and phaeopigments (error bars indicate standard error of total phytopigment contents; n = 3) and (**b**) protein, carbohydrate and lipid concentrations (error bars indicate standard error of biopolymeric C; n = 3). CS = Presence of *C. cylindracea*. NCS = Absence of *C. cylindracea*.

**Figure 2:**
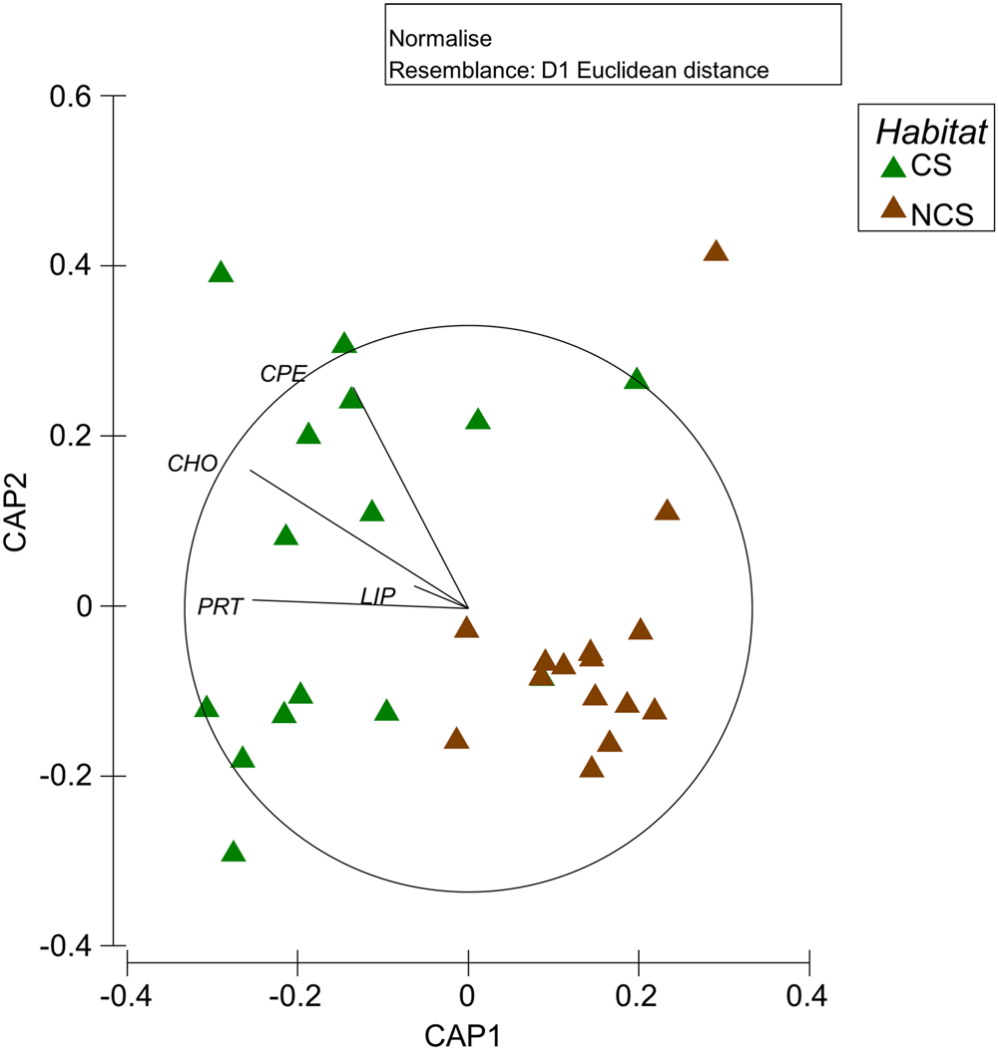
CAP of sedimentary organic matter. Canonical analysis of principal coordinates (CAP) plot showing the discrimination of colonized (CS) and not-colonized (NCS) sediments based on the composition of sedimentary organic matter. Vectors are proportional to the Pearson correlation of the carbon source variables with the ordination axes (for r > 0.75). CPE = chloroplastic pigments; CHO = carbohydrates; PRT = proteins; LIP = lipids; n = 3).

The results of the PERMANOVA tests reveal that the biochemical composition and the contents of each of the investigated variables (with the exception of phytopigments) varied significantly between sediments colonized and not-colonized by the seaweed across locations (Table 1). In all locations, both phytopigment and biopolymeric C contents in sediments colonized (CS) by the seaweed were significantly higher than those in not-colonized sediments (NCS) (Fig. 1a,b). The results of the pairwise comparisons indicate that differences in sedimentary OM composition between sediments colonized and not-colonized by the seaweed were statistically significant at all locations, except for the Bay of Kotor (Table 2). Nevertheless, the CAP plot shows a clear segregation across locations between habitats (Fig. 2), which is mostly explained by the increased contents of phytopigments and carbohydrates in presence of *Caulerpa*. Instead, differences in protein and lipid contents between habitats vary across locations (Tables 1 and 2, Fig. 2).

**Table 1:** Results of PERMANOVA testing for the effects of location and habitat on the biochemical composition of sedimentary OM, phytopigment, protein, carbohydrate and lipid contents.

Source	df	OM composition			Phytopigments			Proteins			Carbohydrates			Lipids		
		MS	Pseudo-F	P	MS	Pseudo-F	P	MS	Pseudo-F	P	MS	Pseudo-F	P	MS	Pseudo-F	P
Lo	4	4.01	2.20		0.24	0.33		0.99	3.60		1.76	9.40		1.02	1.61	
Ha	1	43.67	8.77		11.02	17.55	*	14.46	11.33		14.93	18.27		3.26	1.44	
Lo x Ha	4	4.98	2.74	**	0.63	0.87		1.28	4.65	*	0.82	4.36	*	2.26	3.58	*
Residual	20	1.82			0.73			0.27			0.19			0.63		

df = degree of freedom; MS = mean squares; Pseudo-F = F critic; P (perm) = permutational level of probability. * = P < 0.05; ** = P < 0.01.

**Table 2:** Results of the pairwise tests contrasting OM composition (OM, proteins, carbohydrates and lipids) between colonized (CS) and not-colonized (NCS) sediments within each Location.

Variable	OM composition		Proteins		Carbohydrates		Lipids	
Contrast	CS vs NCS		CS vs NCS		CS vs NCS		CS vs NCS	
	T	P(MC)	T	P(MC)	T	P(MC)	T	P(MC)
Bay of Kotor	1.42	ns	2.43	ns	7.66	**	0.21	ns
Othonoi Island	2.99	*	0.62	ns	3.51	*	0.28	ns
Otranto	2.51	*	4.47	*	5.59	**	5.40	**
Porto Cesareo	2.30	*	5.38	**	3.57	*	0.73	ns
Torre Guaceto	3.09	*	3.03	*	33.97	***	2.41	ns

T = T value, P(MC) = probability level after Monte Carlo simulations. * = P < 0.05; ** = P < 0.01; *** = P < 0.001; ns = not significant.

### Metabolic patterns of microbial communities

Overall, microbes in the three investigated habitats (i.e., AT, CS, and NCS) show the ability to degrade most of the investigated carbon sources, with the exceptions of the aminoacid L-threonine, four carboxylic acids (γ-Hydroxybutyric, D-Glucosaminic, α-Ketobutyric, and D-Malic acids) and α-Cyclodextrin (Supplementary Table S1).

In each location, the highest number of degraded substrates (here used as an estimate of the metabolic capacity of microbial communities) recurs in sediments colonized by the seaweed (Supplementary Table S1).

Metabolized substrates found ubiquitously in all locations and habitats include: L-Phenylalanine, L-Serine, Phenylethyl-amine and Putrescine among amino acids; β-Methyl-D-Glucoside, D-Xylose, D-Cellobiose, Glucose-1-Phosphate, α-D-Lactose and D,L-α-Glycerol Phosphate among carbohydrates; Pyruvic Acid Methyl Ester and D-Galacturonic Acid among carboxylic acids; Tween 40 and Tween 80 among polymers (Supplementary Table S1). All the benthic microbial communities are able to degrade D-Galactonic Acid γ-Lactone and L-Arginine. Only the microbial communities from AT and CS degrade the 4-Hydroxy Benzoic Acid.

Differences in the microbial metabolic patterns and in the microbial Shannon Index across the three habitats (AT, CS, NCS) vary among locations, as evidenced by the significant Lo × Ha interaction term (Table 3).

**Table 3:** Results of PERMANOVA testing for differences in the metabolic pattern of microbial communities and metabolic Shannon Index among habitats in the five sampling locations.

Source	df	Metabolic pattern			Shannon		
		MS	Pseudo-F	P(perm)	MS	Pseudo-F	P(perm)
Lo	4	781.5	18.0		13.67	603.97	
Ha	2	1447.2	4.6		29.37	8.40	
Lo x Ha	8	312.7	7.2	***	3.50	154.54	***
Residual	30	43.4					

df = degree of freedom; MS = mean squares; Pseudo-F = F critic; P(perm) = permutational level of probability. *** = P < 0.001.

**Figure 3:**
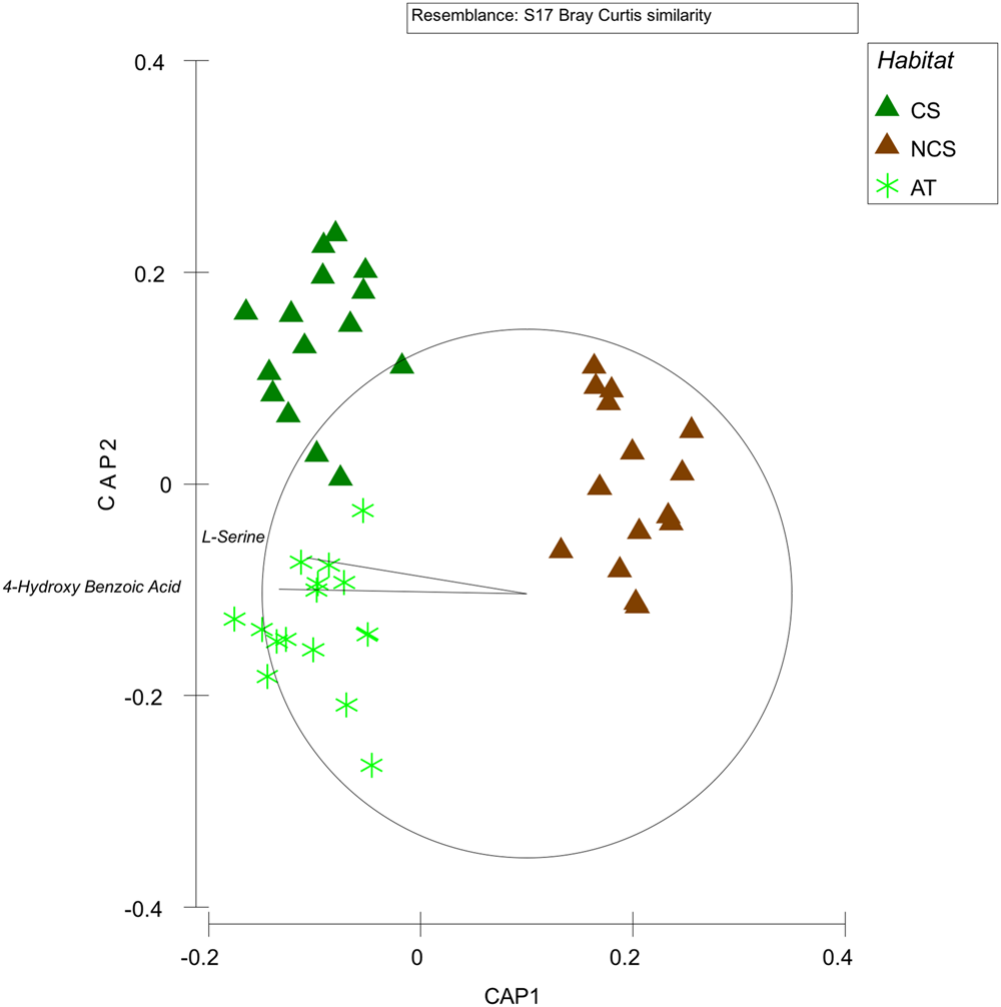
CAP of metabolic patterns of microbial communities. Canonical analysis of principal coordinates (CAP) plot showing canonical axes that best discriminate metabolic patterns of microbial communities hosted on the algal thalli (AT), colonized (CS) and not-colonized (NCS) sediments. Vectors refer to the carbon source variables best correlated with the canonical axes.

Post-hoc pairwise tests carried out separately within each location reveal significant differences among the three habitats in all locations, with the exception of Otranto where no difference was observed between CS and NCS (Table 4). Accordingly, the CAP plot shows a clear segregation of the three habitats, with the highest differences driven by the presence/absence of the seaweed. This analysis also shows that the metabolic patterns of microbial communities hosted on the seaweed are more similar to those in colonized sediments than those in not-colonized sediments. The CAP achieved the highest allocation success using m = 7 principal coordinate axes, which themselves also explained ca. 98% of the variation in the original dissimilarity matrix (Table 5). Pearson correlations coefficients (>0.75) suggest that 4-hydroxy benzoic acid and L-serine mainly characterized the microbial communities on both the *C. cylindracea* thalli (AT) and the sediments colonized by the seaweed (CS) (Fig. 3).

**Table 4:** PERMANOVA pairwise tests contrasting microbial metabolic patterns and Shannon Index between pairs of the three investigated habitats (AT, CS, and NCS) within each Location.

Location	Contrast	Metabolic pattern		Shannon	
		T	P(MC)	T	P(MC)
Bay of Kotor	CS vs NCS	5.35	**	27.01	***
	CS vs AT	3.48	**	17.96	***
	NCS vs AT	4.33	**	6.65	**
Othonoi Island	CS vs NCS	2.91	*	21.25	***
	CS vs AT	2.74	*	6.61	**
	NCS vs AT	4.56	**	17.41	***
Otranto	CS vs NCS	2.33	ns	15.29	***
	CS vs AT	3.22	**	60.15	***
	NCS vs. AT	4.13	**	15.73	***
Porto Cesareo	CS vs. NCS	3.11	*	52.43	***
	CS vs AT	2.23	*	9.74	***
	NCS vs AT	2.77	**	16.49	***
Torre Guaceto	CS vs NCS	2.79	**	10.72	***
	CS vs AT	3.36	**	28.11	***
	NCS vs AT	4.00	**	15.11	***

T = T value, P(MC) = probability level after Monte Carlo simulations. * = P < 0.05; ** = P < 0.01; *** = P < 0.001; ns = not significant.

**Table 5:** Results of leave-one-out allocation success from the canonical analysis of principal coordinates (CAP) carried out on the microbial metabolic patterns in algal thalli (AT), colonized (CS) and not-colonized (NCS) sediments.

A priori group	CS	NCS	AT	Total	% correct
**CS**	14	0	1	15	93
**NCS**	0	15	0	15	100
**AT**	0	0	15	15	100

The analysis was done using the first m = 7 principal coordinate axes (explaining altogether ca. 98% of the variation in the original dissimilarity matrix) based on the Bray-Curtis dissimilarities on untransformed data.

## Discussion

Overall, the results of our study show an increase in the quantity of sedimentary OM in presence of *C. cylindracea* together with changes in organic OM composition, which can support microbial communities able to degrade substrates released by the seaweed.

The biochemical composition of *C. cylindracea* is generally characterised by the dominance of carbohydrates, over proteins, and lipids^36,37^. Accordingly, a consistent dominance of carbohydrates over the other compounds has been observed also in colonized sediments investigated here and elsewhere^23^. In marine sediments, proteins and phytopigments are often used as proxies of the freshness and nutritional quality of sedimentary organic matter^34^, whereas carbohydrates are generally considered the fraction of sedimentary OM less available for heterotrophs^38^. However, the carbohydrates here investigated are pre-eminently derived from the seaweed, as suggested by the higher phytopigment contents associated with *C. cylindracea*. Since marine seaweeds, differently from seagrasses^33^, are an important source of labile (and therefore nutritionally available) carbohydrates for the benthos^39,40^, we suggest that the presence of *C. cylindracea* can also have an effect on the nutritional quality of sedimentary OM. In addition, the observed differences in OM contents in the sediments colonized by the seaweed could be also due to the reduced hydrodynamic force at the sediment surface determined by the presence of the seaweed itself^18^. Altogether our results corroborate previous hypotheses suggesting that the presence of this seaweed can increase the trophic status of colonized sediments^23^, by releasing its remainders and dissolved organic compounds that could serve as a substrate for benthic heterotrophs, including microbes.

We report here that the metabolic profiles of microbial communities vary significantly among the three habitats (i.e. algal thalli, colonized and not-colonized sediments). Although laboratory conditions can be different from field conditions, the adopted method is considered a good proxy to assess the microbial metabolic activities^30,41,42^. Our results show that the metabolic patterns of microbial communities hosted on the seaweed are more similar to those in colonized sediments than those in not-colonized sediments. Based on this result, we suggest that the presence of *C. cylindracea* favours the selection of benthic microbial communities which are specifically able to exploit the organic releases from the seaweed. In turn, these microbial communities could be also involved in the host metabolism. In fact, sediments invaded by *C. cylindracea* are characterised by increased OM contents and accumulation of sulphides which favour the onset of hypoxic conditions^8,21^: this could allow the development of microbial communities that support nitrogen fixation and enhance OM turnover, hence providing nutrient supply to the seaweed itself^43,44^.

The genus *Caulerpa* can be considered as a holobiont created by bacteria-seaweed long-term coevolution, together with sporadic events of lateral transfer between hosted and environmental bacteria^25^. Seaweed-associated prokaryotes could exchange signals with their algal hosts, metabolize algal derived compounds, and synthesize algal hormones^45^. In this regard, we noticed that L-serine is C source more degraded by microbial communities in the sediments colonized by *C. cylindracea* than in sediments not-colonized. L-serine is an important intermediate of photorespiratory glycolate pathway in several marine seaweeds, and the measurement of its formation may be a good indication of the amount of carbon flowing through this pathway^46–48^. Several prokaryotic species belonging to the genus *Shewanella* are able to degrade L-serine and this genus was indeed found on *C. cylindracea* ^27–29^. Based on these insights, we interpret the presence of an active metabolic ability to degrade this aminoacid as an indication of the stimulating effect of *C. cylindracea* on sedimentary microbial communities specifically adapted to the presence of the seaweed. Both microbial communities degraded L- asparagine: it is a component of living organisms utilized by some marine luminous bacteria belonging to *Vibrio* and *Photobacterium* genera, which are known to produce large amounts of L-asparaginase^46^. Both these genera were found on *C. cylindracea* ^27–29^. Given our results, understanding if different metabolic functions driven by seaweeds-bacteria associations actually reflect distinct microbial communities can be an important development of our research to be ascertain with metagenomics^47^.

Both the algal thalli and the sediments colonized by the seaweed were characterised by microbes, otherwise missing in the not-colonized sediments, able to digest 4-Hydroxy Benzoic Acid. Carboxylic acids are major constituents of the pools of organic matter in several aquatic environments^48–50^, and hydroxybenzoic acids, in general, are important intermediate metabolites in the degradation of various aromatic compounds due to some bacterial taxa, including the genus *Bacillus* ^51^. Since we recently isolated bacteria belonging to the genus *Bacillus* from *C. cylindracea* ^27–29^, we conclude that the presence of microbes able to digest 4-Hydroxy Benzoic Acid in the sediments immediately neighbouring the seaweed could represent a tangible sign of the effects of *C. cylindracea* invasion on the functional identity of benthic microbes.

Non-indigenous invasive species can exert major effects on the structure and functions of marine biodiversity^3,4^ and, as a critical consequence, on their ability to provide goods and services^13,52–54^. The presence of the invader affects the local characteristics of the sediment, including the quantity, biochemical composition, and nutritional quality of organic detritus^55^, and, as a consequence, may result in changes in the rate of organic matter decomposition^23,56,57^. In this regard, our results show that the algal thalli and the sediments colonized by the seaweed host microbial communities with spectra of metabolic activity larger than those in sediments where the seaweed is absent. Indeed, in all sampling locations the microbial communities in colonized sediments showed the ability to degrade a higher number of substrates than the one in not-colonized sediments. This result further supports the hypothesis that the presence of *C. cylindracea* can influence the overall functioning of the benthic microbial loop, through changes in the quantity, biochemical composition, and nutritional quality of organic detritus. Although in this study we have not ascertained quantitatively how much this can modify organic matter degradation rates, it is noticeable that C degradation rates in sediments invaded by *C. cylindracea* can be lower than those in not invaded sediments^23^. Thus, although the presence of *C. cylindracea* can stimulate the development of microbial communities specifically adapted to degrade a spectrum of substrates larger than that in not-colonized sediments, we conclude that the overall effect of this seaweed on sedimentary C degradation rates can be a noticeable impact on benthic biogeochemical cycles.

This study represents, to our best knowledge, the first effort to understand the potential effects of *C. cylindracea* on the biogeochemistry of coastal sedimentary habitats through the analysis of the metabolic patterns of benthic microbial communities. Our results allowed us to suggest that seaweed invasions can induce important transformation in sedimentary habitats potentially modifying the supply, quantity and bioavailability of resources as well as benthic biogeochemical cycles. Some studies have demonstrated that *C. cylindracea* acts as a passenger taking advantage of habitat degradation^11–58^. While we are aware that further investigations are needed to clarify the mechanisms by which this invasive seaweed influences microbial populations and their impacts on biogeochemical cycles at larger spatial scales, we document that the presence of this seaweed, besides the effects on benthic biodiversiy^7,59^, could represent a potential driver of change for benthic ecosystems’ functioning. Since warming and the spread of invasive species are affecting marine ecosystems worldwide^60,61^ and since these global change-related disturbances are exacerbated in the Mediterranean Sea^4,62^, we anticipate that a further spreading of *C. cylindracea* could likely have important consequences on the biogeochemical cycles of its coastal areas.

## Material and Methods

### Sampling

**Figure 4:**
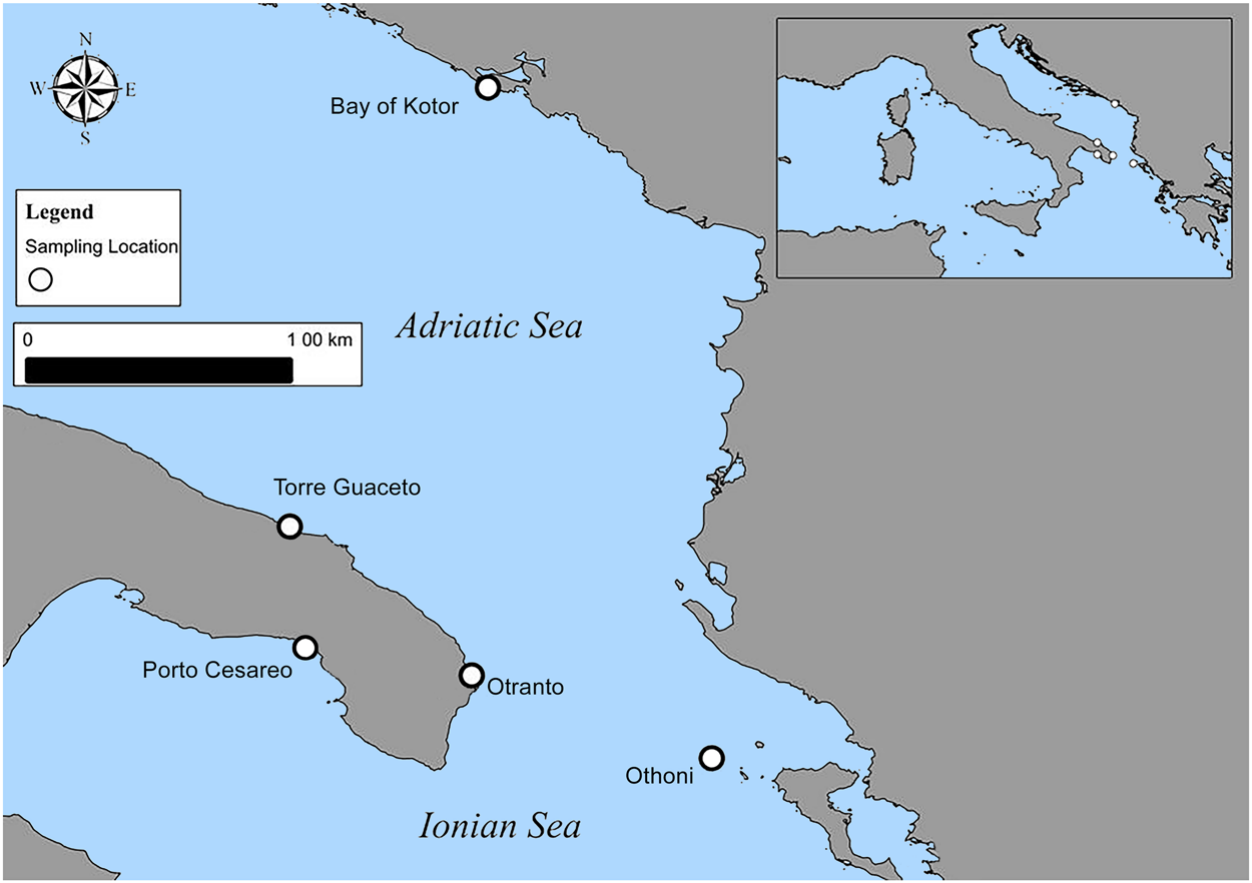
Sampling locations. Map of the five sampling locations in the Mediterranean Sea. The map was created with the Quantum Gis v. 2.18 software (www.qgis.org).

Thalli of the invasive seaweed *C. cylindracea* (AT), along with colonized (CS) and not-colonized (NCS) sediments were collected by SCUBA divers manually and by manual corers, for the seaweed and the sediments respectively, in September 2013 at shallow water (5-10 m depth) in five different locations (Fig. 4): the Bay of Kotor, Montenegro (42°29′06.6″N, 18°41′28.6″E); Othonoi (Diapontine Islands) Greece (39° 50.257′N, 19° 24.037′E); the Marine Protected Area of Torre Guaceto (Brindisi), Italy (40°42′59.25″N, 17°48′5.12″E); Otranto (Lecce), Italy (40° 9′5.94″N, 18°29′27.16″E) and the Marine Protected Area of Porto Cesareo (Lecce), Italy (N 40°12.772′, E017°48.218′). Locations are separated at least by 100 km. CS and NCS differed only in terms of presence/absence of seaweed. Sampling units were randomly collected, about 10 m apart. Once collected, replicated samples of the seaweed and of the sediments were transferred to the laboratory under controlled temperature and processed within 4 h.

### DNA extraction, amplification and sequencing of the seaweed

Molecular analyses were performed to confirm the identification of *C. cylidracea* species. Genomic DNA was isolated by a CTAB DNA extraction method^63^. Amplification by PCR was performed in a master mix of volume 25 µL containing 5 pmol of each primer; 200 µM of each dNTP; 1X assay buffer; and 1.25 units of Taq DNA polymerase. The reactions were exposed to the following PCR profile using the specific primers TufA-F (TGAAACAGAAMAWCGTCATTATGC) and TufA-R (CCTTCNCGAATMGCRAAWCGC)^64^: 35 cycles of denaturation (94 °C for 1 min), primer annealing (50 °C for 1 min), and extension (72 °C for 2 min). A 5-min final extension cycle at 72 °C followed the 40th cycle to ensure the completion of all novel strands. PCR products were purified and subjected to commercial sequencing. The sequences of all isolates were compared with those of closely related sequences available on GenBank. Multiple sequence alignments were performed with CLUSTAL W at the Kyoto University Bioinformatic Center (http://www.genome.jp/tools/clustalw/). The CLUSTAL W output file was used to construct evolutionary tree with the SeaView software^65^ in accordance with the maximum-likelihood method^66^. Tree robustness was assessed by bootstrap resampling (1000 replicates each). Sequences were made available on GenBank database with the accession numbers KY773569–KY773573.

### Biochemical composition of sedimentary organic matter

Chlorophyll-a and phaeopigment analyses were carried out fluorometrically^39^. Pigments were extracted (12 h at 4 °C in the dark) from triplicate superficial (0–1 cm) sediment aliquots (ca. 1 g wet weight), using 5 ml of 90% acetone as the extractant. Extracts were analysed fluorometrically to estimate chlorophyll-a, and, after acidification with 200 µL of 0.1 N HCl, to estimate phaeopigments concentrations. Concentrations are normalised to sediment dry weight and reported as μg g^−1^. Total phytopigments were defined as the sum of chlorophyll-a and phaeopigments^67^. Protein, carbohydrate and lipid contents were analysed spectrophotometrically^39^, and expressed as albumin, glucose and tripalmitine equivalents, respectively. For each biochemical assay, blanks were obtained using pre-combusted sediments (450 °C for 4 h), and analyses performed on triplicate superficial (0–1 cm) samples. Carbohydrate, protein, and lipid contents were converted into carbon equivalents using the conversion factors 0.40, 0.49, and 0.75, respectively, and their sum reported as biopolymeric C^68^.

### BIOLOG ECO plate inoculation and incubation

In the laboratory, fragments of the seaweed and aliquots of the sediments from the top 1 cm of each corer were suspended in sterile seawater and, prior to the analyses, sonicated for three times (Branson Sonifier 2200, 60 W, 47 kHz for 1 min in an ice bath) to optimize prokaryote detachment from their substrate. The sonication was interrupted for 30 s every minute, and, during this interval, the samples were shaken manually.

BIOLOG ECO plate (BIOLOG Inc., Hayward, Calif.) is a system made by a set of 31 carbon substrates and one blank well in triplicate. The substrates include 8 amino acids, 9 carbohydrates, 10 carboxylic and acetic acids and 4 polymers. In each well a volume of 150 µL of a suspension adjusted in order to contain approximately 1 **×** 10^4^ cell ml^−1^ and the BIOLOG ECO plates incubated at 25 °C for 1 week. The optical density (OD) values for each well was measured at a wavelength of 590 nm at the beginning and the end of the incubation with a plate reader, and the resulting variation obtained by subtraction, after removal of eventual fluorescence from the blanks. The increase in OD values was then considered as an indicator of the growth of microbial communities able to degrade the substrate^69^. This method is considered a good indicator to evaluate the microbial metabolic activities in environmental samples, as it reflects changes of metabolic activity and/or potential functional versatility of microbial communities exposed to several stressors^29,42^. It furnishes a proxy of metabolic fingerprint of environmental samples^41^.

### Statistical analyses

The differences in i) the sedimentary OM composition between colonized and not-colonized sediments, and ii) the metabolic patterns of microbial communities associated with the seaweed and those in the colonized and not-colonized sediments were assessed across locations by multivariate analyses.

To assess differences in the metabolic patterns of microbial communities among the algal thalli (AT), colonized (CS) and not-colonized (NCS) sediments the design consisted of two factors: Location (Lo, as random factor with 5 levels) and Habitat (Ha, as fixed factor with 3 levels orthogonal to Lo (separated by about 50–100 m), with n = 3 for each combination of factors (separated by about 10 m). Multivariate (metabolic patterns) and univariate (Shannon Index) analyses of variance (PERMANOVA)^70^ were based on Bray Curtis dissimilarities on untransformed data, using 9,999 random permutations of the appropriate units^71^. For evaluating the allocation success of the observed metabolic patterns to the *a priori* groups (i.e. levels of the factor Habitat: CS, NCS and AT), canonical analysis of principal coordinates (CAP)^72,73^ was performed pooling together data from all locations.

The same design was applied to detect differences in the composition of organic matter between sediments colonized and not-colonized by *C. cylindracea*. The experimental design consisted of two factors: Location (Lo, as random factor with 5 levels) and Habitat (Ha, as fixed factor with 2 levels, i.e., presence/absence of *C. cylindracea*, orthogonal to Lo) with n = 3 for each combination of factors. Multivariate (OM) and univariate (proteins, carbohydrates and lipids) analyses (PERMANOVA)^70^ was based on Euclidean distances of previously normalized data, using 9,999 random permutations of the appropriate units^71^. For illustrating differences in the composition of sedimentary OM, significant terms were plotted using canonical analysis of principal coordinates (CAP)^72,73^ for the factor Habitat (i.e. CS vs NCS).

For both designs, when significant differences were encountered (p < 0.05), post-hoc pairwise tests for the fixed factor were carried out, to ascertain the consistency of the differences among habitats across locations. Because of the restricted number of unique permutations in the pairwise tests, p values were obtained from Monte Carlo samplings. The analyses were performed using the software PRIMER v. 6^74^.

## Electronic supplementary material

Supplementary Information
