# Supplementary material for: Potential effects of an invasive seaweed (Caulerpa cylindracea, Sonder) on sedimentary organic matter and microbial metabolic activities
Source: Sci Rep. 2017 Sep 21;7:12113. doi: 10.1038/s41598-017-12556-4 (PMC5608702; doi:10.1038/s41598-017-12556-4)
Supplement: Supplementary file 1 — Supplementary Information [file 41598_2017_12556_MOESM1_ESM.doc]

**Potential effects of an invasive seaweed (*Caulerpa cylindracea*, Sonder) on sedimentary organic matter and microbial metabolic activities**

Lucia Rizzo^1-2-3*^, Antonio Pusceddu ^2-4^, Loredana Stabili^1-5^, Pietro Alifano^1^, Simonetta Fraschetti^1-2^

^1^ Department of Biological and Environmental Sciences and Technologies, University of Salento, Via Prov.le Lecce Monteroni, Lecce

^2^ CoNISMa, Piazzale Flaminio, 9 – Roma – Italy

^3^ Department of Environmental Sciences, Informatics and Statistics, Ca’ Foscari University of Venice, Italy

^4^ Department of Life and Environmental Sciences, University of Cagliari, Via T. Fiorelli 1, Cagliari, Italy

^5^ Institute for Coastal Marine Environment of the National Research Council, U.O.S. di Taranto, Via Roma 3, Taranto, Italy

** Corresponding author:* lucia.rizzo@unisalento.it

**Supplementary Information**

**Supplementary Table S1**

**Table S1** Carbon sources degraded by microbial communities on the algal thalli (AT), colonized (CS) and not-colonized (NCS) sediments in the five sampling locations. Sources highlighted in light blue are those ubiquitous in all locations and habitats.

|  | Bay of Kotor | | | Othonoi Island | | | Otranto | | | Porto Cesareo | | | Torre Guaceto | | |
| --- | --- | --- | --- | --- | --- | --- | --- | --- | --- | --- | --- | --- | --- | --- | --- |
|  | NCS | CS | AT | NCS | CS | AT | NCS | CS | AT | NCS | CS | AT | NCS | CS | AT |
| **Amino acids** |  |  |  |  |  |  |  |  |  |  |  |  |  |  |  |
| L-Arginine | 1,48±0,02 | 1,11±0,13 | 0±0 | 1,35±0,02 | 1,27±0,17 | 0±0 | 1,89±0,16 | 1,87±0,16 | 1,37±0,11 | 1,97±0,07 | 1,85±0,19 | 1,47±0,19 | 1,64±0,2 | 1,57±0,22 | 1,23±0,19 |
| L-Asparagine | 1,41±0,09 | 0,61±0,04 | 1,61±0,13 | 1,28±0,09 | 1,35±0,14 | 1,51±0,26 | 1,48±0,04 | 1,46±0,04 | 1,31±0,12 | 0±0 | 1,94±0,21 | 2,28±0,04 | 1,43±0,28 | 1,22±0,11 | 1,39±0,29 |
| L-Phenylalanine | 0,78±0,15 | 1,54±0,06 | 1,7±0,14 | 0,66±0,15 | 0,84±0,07 | 1,26±0,15 | 1,49±0,16 | 1,47±0,16 | 1,76±0,22 | 0,98±0,07 | 0,9±0,03 | 1,47±0,21 | 1,36±0,06 | 1,11±0,18 | 1,66±0,15 |
| L-Serine | 0,68±0,13 | 1,51±0,09 | 1,56±0,16 | 0,61±0,09 | 1,69±0,05 | 1,03±0,11 | 0,62±0,09 | 1,6±0,09 | 1,53±0,02 | 0,54±0,07 | 1,65±0,06 | 1,15±0,13 | 0,98±0,19 | 1,85±0,07 | 1,9±0,12 |
| L-Threonine | 0±0 | 0±0 | 0±0 | 0±0 | 0±0 | 0±0 | 0±0 | 0±0 | 0±0 | 0±0 | 0±0 | 0±0 | 0±0 | 0±0 | 0±0 |
| Glycyl-L-Glutamic Acid | 1,6±0,04 | 1,15±0,06 | 1,89±0,18 | 0±0 | 0±0 | 0±0 | 1,25±0,06 | 0,47±0,07 | 0±0 | 0±0 | 0±0 | 0±0 | 2,52±0,12 | 0,48±0,03 | 0±0 |
| Phenylethyl-amine | 1,25±0,02 | 1,22±0,27 | 1,43±0,29 | 1,12±0,02 | 1,29±0,12 | 1,77±0,11 | 1,58±0,02 | 1,56±0,02 | 1,86±0,15 | 1,91±0,2 | 1,98±0,13 | 1,05±0,03 | 1,41±0,11 | 1,69±0,14 | 0,83±0,12 |
| Putrescine | 1,81±0,16 | 1,4±0,15 | 2,24±0,21 | 1,69±0,16 | 1,39±0,48 | 1,82±0,22 | 2,31±0,07 | 2,29±0,07 | 1,75±0,51 | 2,24±0,09 | 2,14±0,01 | 2,11±0,09 | 2,66±0,05 | 2,2±0,09 | 1,85±0,44 |
| **Carbohydrates** |  |  |  |  |  |  |  |  |  |  |  |  |  |  |  |
| β-Methyl-D-Glucoside | 1,26±0,06 | 1,48±0,09 | 1,84±0,16 | 1,14±0,05 | 1,26±0,07 | 1,56±0,13 | 1,58±0,06 | 1,56±0,06 | 2,09±0,17 | 2,16±0,19 | 1,77±0,16 | 1,55±0,35 | 1,71±0,15 | 1,65±0,08 | 2,09±0,11 |
| D-Xylose | 1,5±0,01 | 1,6±0,03 | 1,25±0,3 | 1,37±0,01 | 1,49±0,02 | 1,93±0,18 | 1,54±0,05 | 1,52±0,05 | 1,76±0,08 | 1,76±0,08 | 1,7±0,02 | 1,99±0,18 | 1,72±0,06 | 1,7±0,07 | 2,18±0,06 |
| i-Erythritol | 0,51±0,06 | 0,92±0,16 | 0±0 | 0±0 | 0±0 | 0±0 | 0,64±0,11 | 1,26±0,09 | 0±0 | 0±0 | 0±0 | 0±0 | 0,45±0,03 | 1,47±0,04 | 0±0 |
| D-Mannitol | 0±0 | 1,32±0,07 | 1,15±0,41 | 0±0 | 0,58±0,08 | 1,47±0,23 | 1,48±0,2 | 1,46±0,2 | 0±0 | 0±0 | 0,74±0,06 | 0±0 | 1,66±0,18 | 1,56±0,09 | 0±0 |
| N-Acetyl-D-Glucosamine | 0±0 | 1,42±0,15 | 0±0 | 0±0 | 0±0 | 0±0 | 0±0 | 0±0 | 0±0 | 0±0 | 0±0 | 0±0 | 0±0 | 0±0 | 0±0 |
| D-Cellobiose | 1,36±0,16 | 1,46±0,05 | 1,4±0,26 | 1,23±0,16 | 1,52±0,04 | 1,55±0,28 | 1,52±0,15 | 1,5±0,15 | 2,14±0,14 | 1,22±0,26 | 1,1±0,06 | 1,12±0,07 | 2,07±0,1 | 1,68±0,04 | 2,17±0,15 |
| Glucose-1-Phosphate | 1,53±0,09 | 1,55±0,02 | 1,54±0,14 | 1,40±0,09 | 1,46±0,01 | 1,78±0,14 | 1,2±0,13 | 1,18±0,13 | 1,91±0,1 | 1,81±0,1 | 1,66±0,09 | 1,57±0,24 | 1,43±0,09 | 1,57±0,05 | 2,1±0,14 |
| α-D-Lactose | 1,91±0,06 | 1,19±0,22 | 1,4±0,22 | 1,78±0,06 | 1,82±0,04 | 2,25±0,04 | 1,86±0,02 | 1,85±0,02 | 2,15±0,1 | 2,29±0,03 | 2,02±0,18 | 2,74±0,32 | 2,14±0,08 | 1,97±0,08 | 2,69±0,11 |
| D,L-α-Glycerol Phosphate | 1,52±0,06 | 1,3±0,32 | 1,56±0,17 | 1,39±0,06 | 1,53±0,13 | 2,3±0,13 | 1,51±0,11 | 1,49±0,11 | 2,26±0,13 | 2,1±0,15 | 2,23±0,14 | 1,77±0,44 | 2,05±0,21 | 1,93±0,11 | 2,27±0,12 |
| **Carboxylic Acid** |  |  |  |  |  |  |  |  |  |  |  |  |  |  |  |
| D-Galactonic Acid γ-Lactone | 1,88±0,21 | 1,37±0,06 | 0±0 | 1,74±0,20 | 1,47±0,2 | 1,61±0,48 | 1,97±0,11 | 1,96±0,11 | 1,9±0,1 | 2,22±0,18 | 2,26±0,1 | 2,4±0,3 | 2,04±0,13 | 2,18±0,27 | 2,04±0,27 |
| Pyruvic Acid Methyl Ester | 0,79±0,04 | 1,36±0,11 | 1,6±0,13 | 0,67±0,04 | 1,06±0,16 | 1,84±0,46 | 1,58±0,15 | 1,56±0,15 | 1,56±0,23 | 1,93±0,44 | 1,71±0,08 | 2,43±0,12 | 1,68±0,18 | 1,28±0,21 | 1,73±0,09 |
| D-Galacturonic Acid | 1,16±0,14 | 1,57±0,06 | 1,57±0,22 | 1,03±0,14 | 1,12±0,23 | 1,55±0,16 | 1,66±0,04 | 1,64±0,04 | 1,65±0,12 | 1,89±0,13 | 1,63±0,18 | 2,2±0,08 | 1,55±0,24 | 1,52±0,1 | 1,45±0,08 |
| 2-Hydroxy Benzoic Acid | 0±0 | 0,51±0,03 | 1,52±0,04 | 0±0 | 0,48±0,01 | 1,15±0,04 | 0±0 | 0±0 | 0±0 | 0±0 | 0±0 | 0±0 | 0±0 | 0±0 | 0±0 |
| 4-Hydroxy Benzoic Acid | 0±0 | 1,72±0,02 | 1,1±0,15 | 0±0 | 1,4±0,11 | 1,78±0,09 | 0±0 | 1,6±0,26 | 1,65±0,11 | 0±0 | 1,78±0,2 | 1,66±0,43 | 0±0 | 1,43±0,22 | 2,06±0,12 |
| γ-Hydroxybutyric Acid | 0±0 | 0±0 | 0±0 | 0±0 | 0±0 | 0±0 | 0±0 | 0±0 | 0±0 | 0±0 | 0±0 | 0±0 | 0±0 | 0±0 | 0±0 |
| D-Glucosaminic Acid | 0±0 | 0±0 | 0±0 | 0±0 | 0±0 | 0±0 | 0±0 | 0±0 | 0±0 | 0±0 | 0±0 | 0±0 | 0±0 | 0±0 | 0±0 |
| Itaconic Acid | 0±0 | 0,61±0,09 | 1,27±0,13 | 0±0 | 0±0 | 0±0 | 0±0 | 0±0 | 0±0 | 0±0 | 0±0 | 0±0 | 0±0 | 0±0 | 0±0 |
| α-Ketobutyric Acid | 0±0 | 0±0 | 0±0 | 0±0 | 0±0 | 0±0 | 0±0 | 0±0 | 0±0 | 0±0 | 0±0 | 0±0 | 0±0 | 0±0 | 0±0 |
| D-Malic Acid | 0±0 | 0±0 | 0±0 | 0±0 | 0±0 | 0±0 | 0±0 | 0±0 | 0±0 | 0±0 | 0±0 | 0±0 | 0±0 | 0±0 | 0±0 |
| **Polymers** |  |  |  |  |  |  |  |  |  |  |  |  |  |  |  |
| Tween 40 | 1,25±0,08 | 0,93±0,14 | 1,32±0,11 | 1,12±0,08 | 1,28±0,09 | 1,66±0,1 | 1,6±0,1 | 1,58±0,1 | 1,98±0,1 | 1,38±0,18 | 1,73±0,05 | 1,39±0,2 | 1,74±0,14 | 1,77±0,13 | 1,34±0,18 |
| Tween 80 | 1,4±0,07 | 1,81±0,02 | 1,58±0,04 | 1,27±0,07 | 1,78±0,02 | 1,53±0,04 | 1,67±0,02 | 1,65±0,02 | 1,94±0,06 | 1,81±0,1 | 1,85±0,04 | 2,24±0,07 | 1,78±0,09 | 1,74±0,11 | 2,09±0,05 |
| α-Cyclodextrin | 0±0 | 0±0 | 0±0 | 0±0 | 0±0 | 0±0 | 0±0 | 0±0 | 0±0 | 0±0 | 0±0 | 0±0 | 0±0 | 0±0 | 0±0 |
| Glycogen | 0±0 | 0,88±0,11 | 0±0 | 0±0 | 0±0 | 0±0 | 0±0 | 0,57±0,15 | 0±0 | 0±0 | 0±0 | 0±0 | 0±0 | 0±0 | 0±0 |
| **Total degraded sources** | 19 | 25 | 20 | 17 | 20 | 19 | 20 | 22 | 18 | 16 | 19 | 18 | 20 | 21 | 18 |

**Supplementary Figure S1**

**
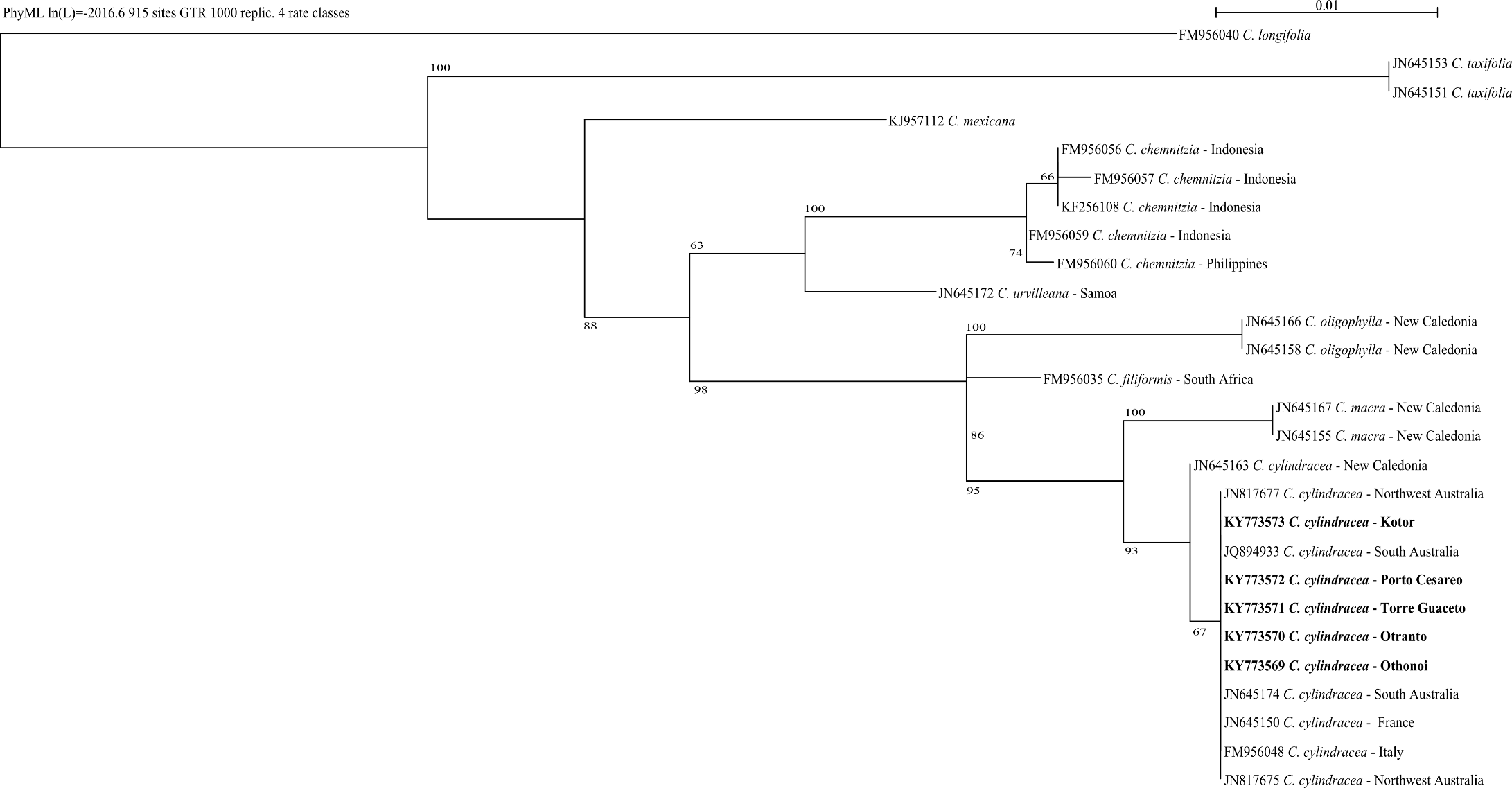
**

**Figure S1** Maximum-likelihood phylogenetic tree based on the *tufA* showing the positions of *Caulerpa cylindracea* sampled in five locations. Bootstrap values (expressed as percentages of 1000 replicates) of >60 % are shown at branch point.
